# Supplementary material for: Modified mRNA-Mediated CCN5 Gene Transfer Ameliorates Cardiac Dysfunction and Fibrosis without Adverse Structural Remodeling
Source: Int J Mol Sci. 2024 Jun 6;25(11):6262. doi: 10.3390/ijms25116262 (PMC11172546; doi:10.3390/ijms25116262)
Supplement: Supplementary file 1 [file ijms-25-06262-s001.zip › ijms-3010136-supplementary.pdf]

Supplementary Figure S1. Representative MRI images and qRT-PCR data for therapeutic intervention model.

A. Representative MRI figure for all three groups; Sham, modRNA-Con, and mod-*CCN5*. The green line shows the epicardial area, the red line indicates the endocardial boundary, and the lime color indicates the infarct area.

B. Representative qRT-PCR results for therapeutic intervention effects in MI-induced HF mouse model. Synthesized cDNAs were used to analyze the mRNA expression level *Tnf-a*, *Mcp-1*, *Il6*, *Il10*, *Tgf-β1*, *α-Sma*, *Mmp2*, *Fap*, and *Bnp* by qRT-PCR. n = 4 for each experimental group, * $p < 0.05$, ** $p < 0.01$, *** $p < 0.001$.

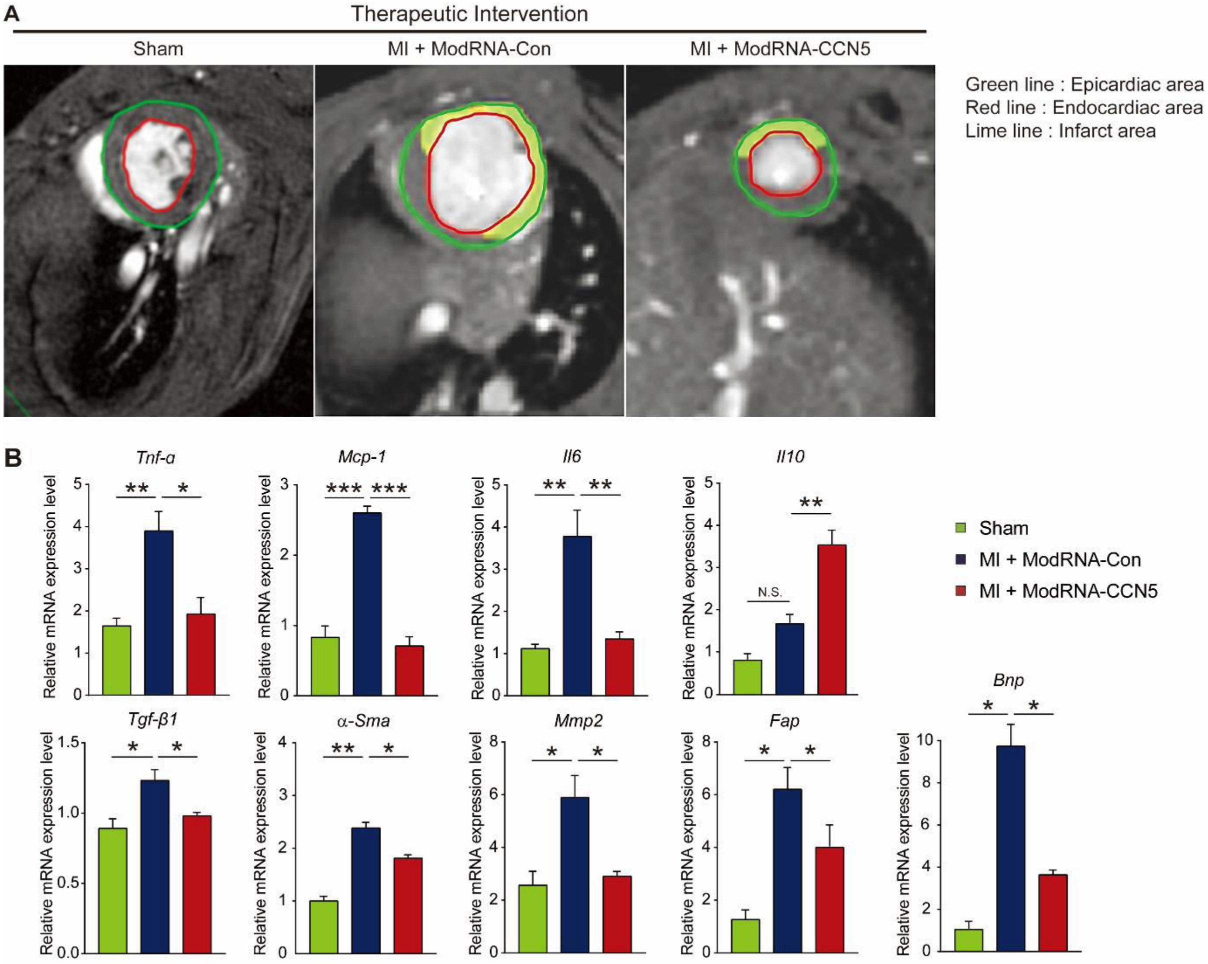

Supplementary Table S1. qRT-PCR primer sequences.

**Gene symbol**	**Primer sequence (5´- 3´)**
Tgf-β1	F: CAACAATTCCTGGCGTTACC<br>R: GAAAGCCCTGTATTCCGTCT
Il6	F: CAA AGC CAG AGT CCT TCA GAG<br>R: GTC CTT AGC CAC TCC TTC TG
Tnf-α	F: ACA GAT GAC ATG GTG AAG ACG<br>R: TCG TTC TTG TGT AGT TCC AGT G
Mcp1	F: TCTCTTCCTCCACCACTATGCA<br>R: GGCTGAGACAGCACGTGGAT
Il10	F: AGC CGG GAA GAC AAT AAC TG<br>R: GGA GTC GGT TAG CAG TAT GTT G
Mmp2	F: CATGCGGAAGCCAAGATGTG<br>R: GTTTCAGGGTCCAGGTCAGG
α-Sma	F: GTTCAGTGGTGCCTCTGTCA<br>R: ACTGGGACGACATGGAAAAG
Fap	F: CGCGTAACACAGGATTCACT<br>R: TCGGAGGAGAGTTTCCAATG
Bnp	F: GAGGTCACTCCTATCCTCTGG<br>R: GCCATTTCCTCCGACTTTTCTC
Gapdh	F: CCATCAACGACCCCTTCATT<br>R: GACCAGCTTCCCATTCTCAG
