# Supplementary material for: Modified mRNA-Mediated CCN5 Gene Transfer Ameliorates Cardiac Dysfunction and Fibrosis without Adverse Structural Remodeling
Source: Int J Mol Sci. 2024 Jun 6;25(11):6262. doi: 10.3390/ijms25116262 (PMC11172546; doi:10.3390/ijms25116262)
Supplement: Supplementary file 1 [file ijms-25-06262-s001.zip › MRI movies/MRI video clips for the therapeutic intervention.pptx]

## Slide 1
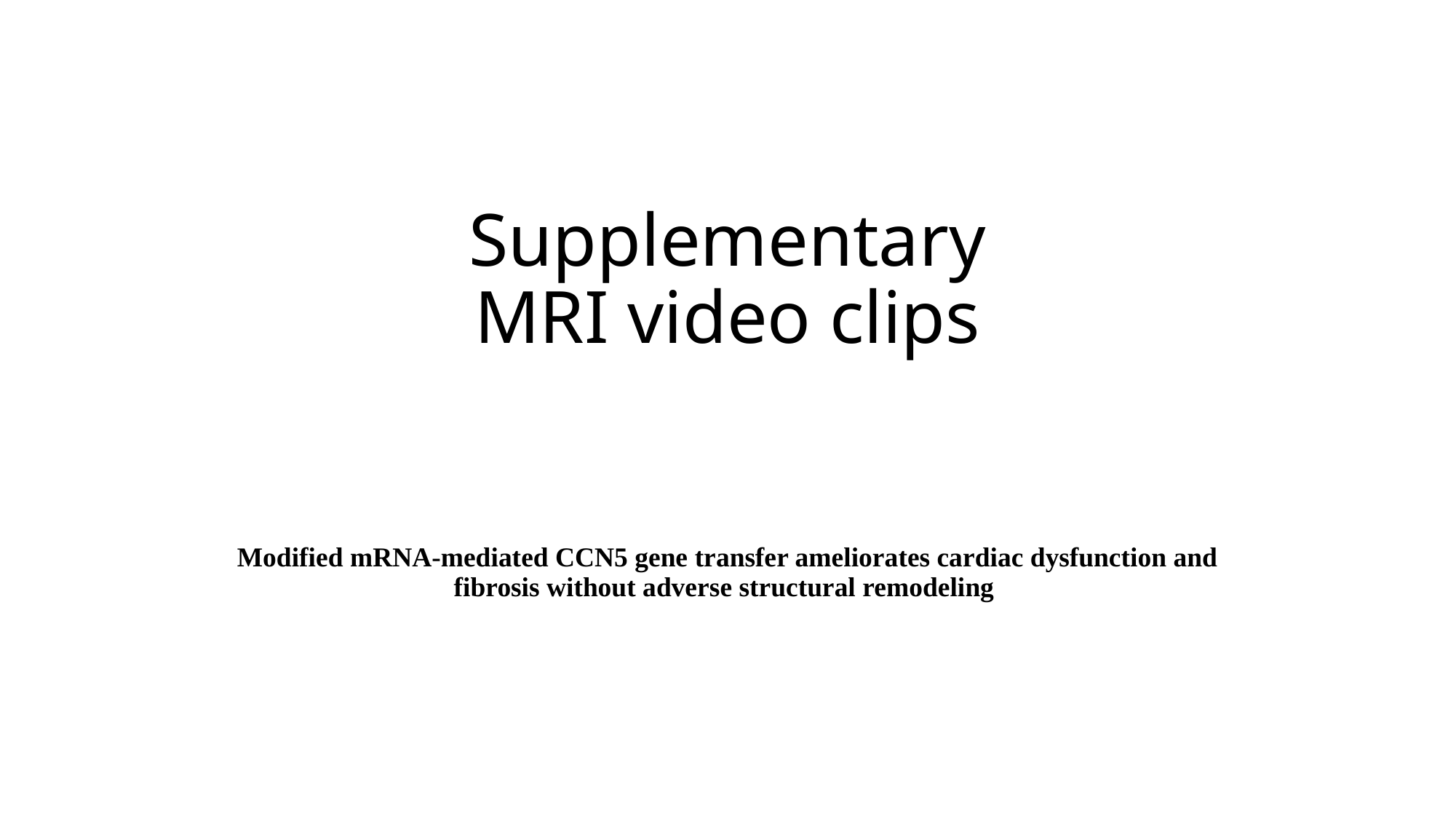

# Supplementary MRI video clips
Modified mRNA-mediated CCN5 gene transfer ameliorates cardiac dysfunction and fibrosis without adverse structural remodeling

## Slide 2
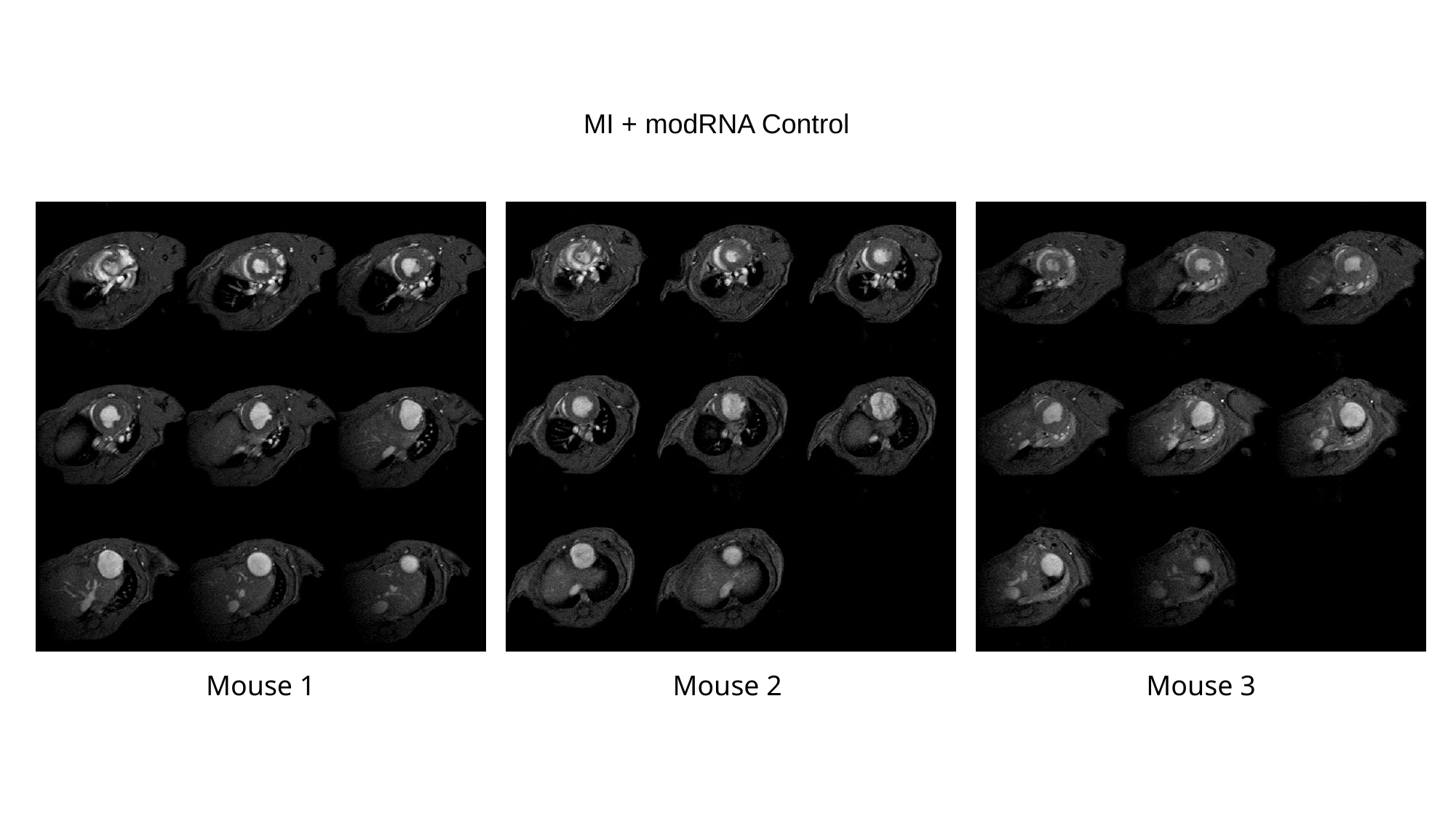

MI + modRNA Control
Mouse 1
Mouse 2
Mouse 3

## Slide 3
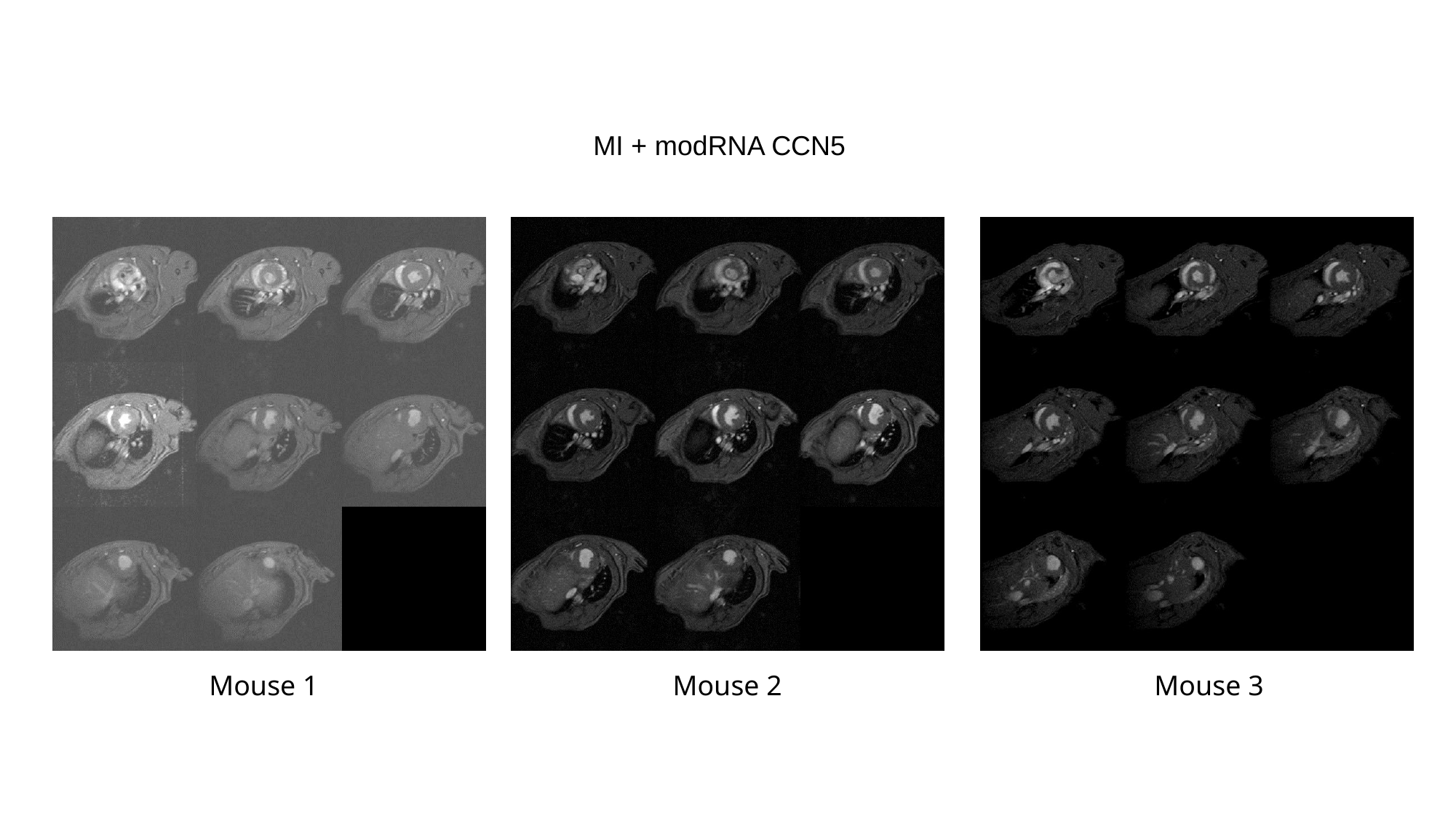

MI + modRNA CCN5
Mouse 1
Mouse 2
Mouse 3
